# Lipidomic Profiling: A Promising New Direction in the Diagnosis and Management of Thyroid Cancer?

**DOI:** 10.7759/cureus.91345

**Published:** 2025-08-31

**Authors:** Valentin Daniealopol, Ruxandra Daniealopol, Tatiana Daniela Sala, Radu Neagoe

**Affiliations:** 1 Surgery, George Emil Palade University of Medicine, Pharmacy, Science, and Technology of Targu Mures, Targu Mures, ROU; 2 Surgery, Emergency Clinical County Hospital Targu Mures, Targu Mures, ROU; 3 2nd Department of Surgery, George Emil Palade University of Medicine, Pharmacy, Science, and Technology of Targu Mures, Targu Mures, ROU

**Keywords:** biomarkers, differentiated thyroid carcinoma, lipidomics, lipid profile, thyroid cancer

## Abstract

Thyroid carcinoma (TC) is the most common endocrine malignancy worldwide, with an ongoing rise in its incidence. Despite improved diagnostic and therapeutic methods, distinguishing between benign and malignant nodules and predicting disease aggressiveness remains challenging. Lipidomics is a comprehensive approach to lipid profiling that is able to provide new insights into thyroid cancer biology. We conducted a methodical literature search for studies that looked into lipid metabolism alterations in the biofluids and tissue samples of TC patients and investigated the potential of the lipidomic fingerprint as a diagnostic or therapeutic target. Studies not employing lipidomic techniques, those using animal models, or focusing on other cancer types were excluded. The reviewed studies consistently revealed significant alterations in various lipid classes, including fatty acids (FA), phospholipids (PL), and sphingolipids (SL) across different sample types (serum, plasma, urine, and tissue) from TC patients compared to benign or healthy controls. Clinically, these findings provide a foundation for more accurate, non-invasive diagnostic tools and for classifying disease subtypes based on lipidomic signatures. For instance, modified glycerophospholipid (GPL) and SL species were observed in the plasma of thyroid cancer patients, and enhanced FA metabolism was correlated with tumor aggressiveness and poor prognosis. Lipidomics is a rapidly evolving field with tremendous potential for improving the clinical management of differentiated TC patients, from diagnosis to therapeutic intervention. The consistent identification of modified lipid profiles highlights their essential role in the metabolic reprogramming associated with tumorigenesis and also their importance as reliable clinical biomarkers. In order to implement routine clinical use of lipidomics, further large-scale validation studies are needed, along with standardized lipidomic protocols, to ensure reproducibility.

## Introduction and background

In the past decades, thyroid carcinoma (TC) has not only shown a rising incidence globally, but has also become the most frequent type of endocrine cancer, making it a significant area in oncological research [1,2]. While current diagnostic and treatment strategies have improved the outcome and prognosis for these patients, the exact cause and complex mechanisms leading to thyroid cancer are not yet completely understood.

Currently, thyroid ultrasonography and fine-needle aspiration biopsy (FNAB) are widely used diagnostic tools that undoubtedly have made a great difference for both clinicians and patients [3]. However, we must admit that they have certain limitations, and it can sometimes be difficult to differentiate between benign and malignant nodules, even by an experienced examiner. Moreover, predicting disease aggressiveness or recurrence risk remains a significant challenge with current diagnostic methods.

Considering these aspects, it is understandable that the role of lipids in cellular signaling and metabolic pathways is becoming a critical area of research. Lipidomics can be defined as a comprehensive analysis of lipidic compounds, as well as their interactions with other molecules, within biological systems [4]. It has recently emerged as a powerful research tool in the oncology field, allowing for an in-depth analysis of the entirety of lipidic compounds in a cell, tissue, or living organism [5,6]. Changes in lipid metabolism were linked to several types of cancer, influencing essential processes such as intercellular communication or cell proliferation [6]. Therefore, lipidomics has drawn significant attention in recent years due to its potential implications in the diagnostic, risk stratification, and therapeutic management of thyroid cancer patients.

The aim of this systematic review is to explore the current state of research and synthesize findings from published papers on the role of lipidomics in TC diagnosis. By understanding the complex interactions between tumorigenesis and lipid metabolism, we seek to unravel new insights into tumor development and progression and to identify potential therapeutic targets eventually.

## Review

Materials and methods

**Figure 1:**
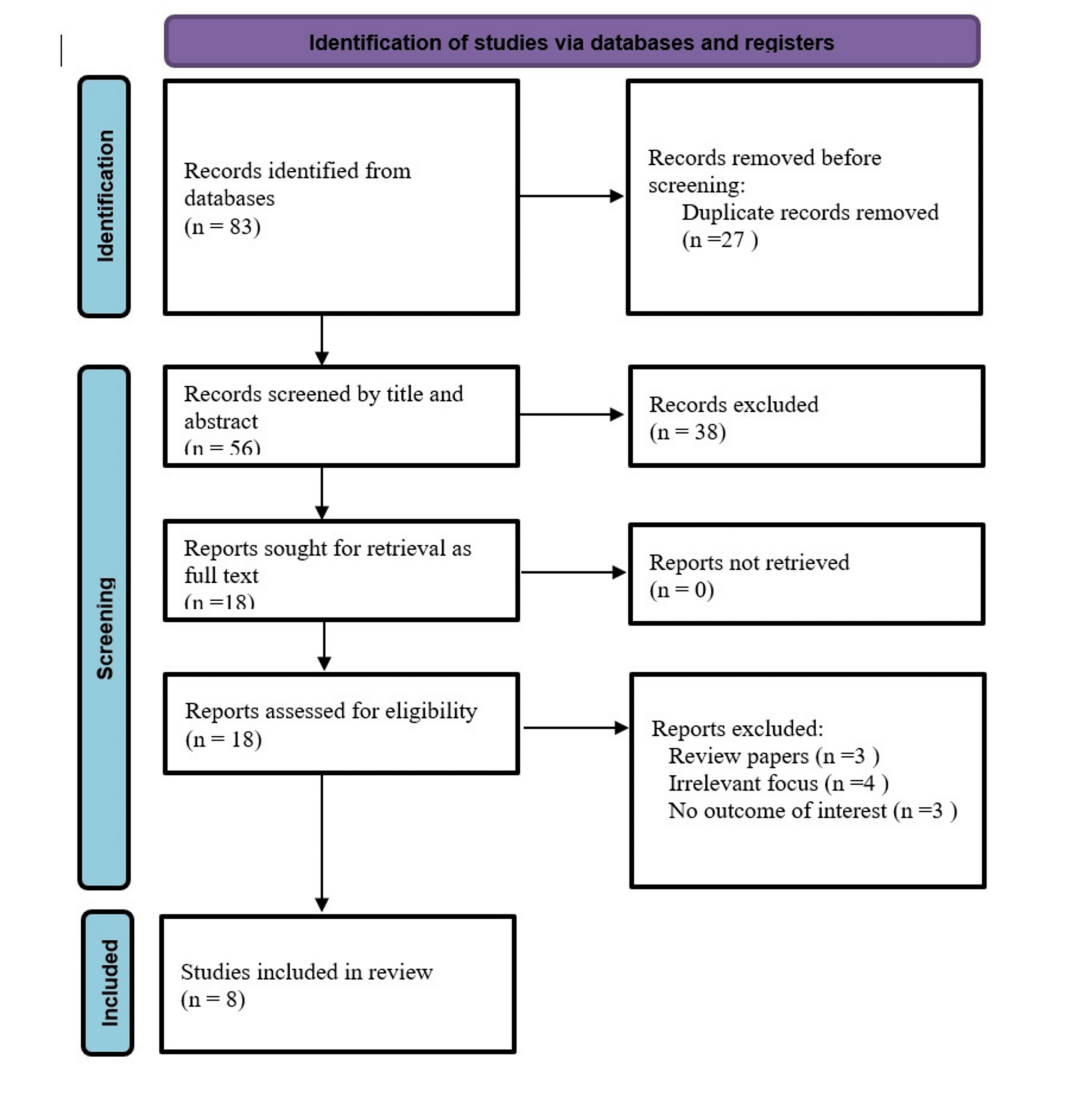
Literature search flow chart

We conducted a systematic literature search on PubMed, Scopus, and Cochrane Library electronic databases using the following keywords: "lipidomics" AND "thyroid cancer" OR "thyroid carcinoma" OR "thyroid neoplasm". We only selected studies published in Romanian and English that were available in full text. The inclusion criteria consisted of clinical research papers, investigating changes in lipid metabolism at the level of thyroid cancer cell lines, tissue samples, or biofluids, published in English or Romanian, available in full text, and conducted on human subjects. The exclusion criteria were: studies that did not use lipidomic techniques, research conducted on animal models, studies concerning other types of cancer, and studies published in languages other than English and Romanian, and publications not available in full text. From each study included, we collected the following information: design of the study, investigation method, sample type, and identified lipid alterations. Both data extraction and quality assessment were performed by two independent reviewers and then reviewed by a third senior researcher. After screening the titles and the abstracts for relevance, we identified eight studies that met the inclusion criteria, published between 2012 and 2024, that were retrieved in full text and submitted to the reviewing process (Figure 1).

Results

Lipidomics and Cancer Research

Several approaches have been successfully implemented in the field of lipidomics research, but the most widely used technique is mass spectrometry (MS)-based lipidomics. This is because it offers both high sensitivity and specificity for identifying an extensive range of molecules [7,8]. Different MS platforms, combined with different ionization and separation techniques, have made it possible to obtain detailed characterization of the lipidome.

Another investigation method currently used is imaging mass spectrometry (IMS), which is particularly useful for better understanding tumor heterogeneity and identifying possible biomarkers [9]. The technique is capable of showing the spatial distribution of lipids within tissue sections, thereby offering valuable insights into the localization of particular lipidic molecules at the level of the tumor microenvironment [10,11].

Regardless of the method of choice, lipidomics analysis will generate complex datasets that will require equally advanced bioinformatics and biostatistical tools for processing, analyzing, and interpreting these data. These sophisticated tools are crucial for identifying changes in lipid profiles and subsequently correlating them with clinical outcomes.

The Role of Lipids in Thyroid Tumorigenesis

Apart from their structural role in cellular membranes, lipids play an important part in numerous signaling pathways involved in processes such as cell proliferation, differentiation, and apoptosis [12]. It is a known fact that the thyroid gland plays an essential part in metabolic regulation and its hormones have a particular influence on lipid metabolism. These complex interactions suggest a potential correlation between altered lipid metabolism and thyroid tumorigenesis [4,5,13].

The potential of metabolomics, particularly lipidomics, in thyroid cancer research was anticipated by scientists almost a decade ago [9,13]. Despite this early recognition, the number of published lipidomic studies remains limited, with many involving small patient cohorts. Researchers have looked into lipid profile changes in bodily fluids and TC tissue compared to normal thyroid tissue. Dysregulation in various types of lipids has been identified, including FA, PL, and SL [9,12,14,15].

Lipidomic Window and Differentiated Thyroid Carcinoma

Wang et al. performed a comprehensive metabolomic and lipidomic analysis on plasma samples collected from 94 patients with papillary thyroid carcinoma (PTC) and 100 controls using ultra-performance liquid chromatography (UPLC)-MS. The integrated metabolomic profiling revealed significant dysregulation in the cancer group compared to healthy controls. The identified modifications indicated that the energy production mechanisms within cancer cells were affected. Moreover, a wide range of lipid metabolism pathways were affected, comprising 12 distinct lipid classes, suggesting there are notable changes in cellular membrane composition and energy storage. In the study, 113 metabolites and 236 differential lipids (out of which 207 had increased and 29 had decreased levels) were identified as statistically significant. When compared to healthy controls, PTC patients presented elevated triacylglycerides, sphingomyelin (SM), phosphatidylethanolamine (PE), phosphatidic acid (PA), lysophosphatidic ethanolamine (LPE), diacylglycerol, ceramide (CER), and cholesterol ester, while acylcarnitine and FA had decreased levels; this indicates an increased metabolism, along with diminished FA synthesis and beta-oxidation. These findings collectively establish a distinct metabolic and lipidomic signature for PTC, moreover revealing several potential targets, such as L-glutamine, indole-3-carboxyaldehyde, or sebacic acid (these showed high sensitivity and specificity in both the discovery and the validation cohort) for both early diagnosis and development of novel therapies [14].

We found another study [16] that investigated the potential of urine metabolic profiling in differentiating between malignant and benign thyroid nodules, and healthy controls. The purpose of this research was to identify specific urinary biomarkers and modified metabolic pathways that could facilitate a non-invasive diagnosis. Urine samples were collected preoperatively from 30 TC patients, 30 benign nodule (B) patients, and 20 healthy controls (C). Both targeted and semi-targeted metabolomic protocols were applied for the analysis of the samples and a statistical model was successfully developed, differentiating between TC, B, and C groups. The model was built upon the identification of 190 different metabolites and then compared with serum metabolites. Increased values of taurocholic acid and decreased levels of docosahexaenoic, linoleic, and mevalonic acids were observed in the TC group. As far as the specific metabolic pathways are concerned, steroid hormone metabolism has been shown to have the highest impact. The lipid metabolites with the best discriminatory power were the following: lysoPE (22:6), mevalonic acid, dihydrocortisol, glycerophosphocholine, androsterone, lysoPE 22:0, 19-norandrosterone, tetrahydrocortisone and lysophosphatidylcholine (lysoPC) (18:3). The lipidomic window proved to be the most relevant for identifying urinary biomarkers that could discriminate between thyroid neoplasms and benign nodules, whereas in blood, the products of amino acid metabolism showed higher sensitivity/specificity relevance. Several unsaturated free FA levels were increased in TC and B patients compared to the C group, with two exceptions - palmitic and myristic acid - thus helping to discriminate between malignant and benign nodules. Also, bile acid metabolism is significantly modified, with elevated levels of keto-, cheno-deoxycholic, and lithocholic acid in the TC group compared to the other two. Prostaglandins (PG) have shown the ability to distinguish between B and TC groups, particularly PGA1 and PGF1a, which were increased in the latter, whereas steroids exhibited modified levels in both B and TC patients compared to healthy controls. The study particularly highlighted the role of lipids and selenium-related molecules in the differential diagnosis, therefore offering a promising new direction for a non-invasive diagnostic strategy.

The same research group published another study [17], analyzing blood samples (serum) of patients with PTC and benign nodules and comparing them to healthy individuals. Using untargeted serum metabolomic profiling, they successfully managed to select 166 distinct relevant metabolites, belonging to 10 different biochemical classes. Statistically significant differences in these metabolic profiles were found when comparing PTC patients to healthy controls, as well as when comparing B patients to the C group. Additionally, differences were observed between the TC and B groups, indicating a unique metabolic signature for each condition: stearic acid (C18:0), PGE2/D2, dimethyl-PGE2, 7-methyl-cholic acid, and lysoPC (20:3) were significantly decreased, whereas arachidonic acid (C20:4), eicosadienoic acid (C20:2), PGE3/D3, lysoPA (18:2), lysoPA (22:1), lysoPA (20:4), and lysoPC (20:4) were significantly decreased in PTC versus B group, with p<0.05. The diagnostic power of these possible biomarkers was also evaluated and revealed that lysoPE (20:4), taurocholic acid, tetrahydrocortisol, lysoPE (22:5), PGF1a, lysoPE (22:2), and dihydrocortisol had higher sensitivity to differentiate between PTC and healthy controls, while lysoPA (18:2), lysoPE (20:4), stearic acid (C18:0), arachidonic acid (C20:4), PGE2/D2, 7-methyl-cholic acid, lysoPC (20:4), and dimetyl-PGE2 were most suitable to discriminate between malignant and benign nodules, according to the area under the curve (AUROC) values.

The metabolic pathways were also analyzed and linoleic and alpha-linolenic acid metabolism was ranked second, respectively seventh out of 19, based on the way they were impacted by thyroid disease. Similar to the previous study [16], where urine samples were used for metabolomic analysis, there is a superior discriminatory power of the lipidomic window. This suggests a complex and distinct metabolic rearrangement, where lipid species and their associated metabolic pathways are most affected by the malignant/benign thyroid nodule development. The study concludes that serum metabolomics analysis can effectively differentiate between PTC, benign thyroid nodules, and healthy controls, thus offering an extremely valuable metabolic overview of thyroid pathophysiology, highlighting the role of lipids and selenium-related molecules as discriminative biomarkers [17].

In a study published in 2021, Jiang et al. suggested that plasma lipidomic profiling could offer a non-invasive diagnosis alternative for PTC. They performed lipidomic analysis on blood samples (plasma) from 47 TC patients and 33 healthy controls. The results showed that linoleic and alpha-linoleic acid metabolism, glycerophospholipid (GPL) metabolism, PI signaling system, and arachidonic acid metabolism were modified in PTC. The ultra-performance liquid chromatography coupled with quadrupole time-of-flight (UPLC-Q-TOF) MS-based analysis was able to identify 13 different metabolites, all belonging to either GPL or SL class, with statistically significant modifications (p<0.05) in their abundance between the PTC group and the control group; the modified metabolites were the following: PG (17:0/14:1), PE (16:0/20:2), PE (P-18:0/18:2), PE (O-18:0/20:5), SM (d18:1/15:0), PE (O-18:0/18:3), SM (d18:1/16:1), PS (20:3/18:0), GlcCER (d14:1/24:1), PC (O-14:0/15:0), SM (d16:1/24:1), PE-NME (18:1/18:1), and phosphatidylserine (PS) (20:4/18:0). After a rigorous screening process, the study managed to narrow down these findings to what appears to be five strong potential biomarkers: GlcCER (d14:1/24:1), PE-NMe (18:1/18:1), SM (d16:1/24:1), SM (d18:1/15:0) and SM (d18:1/16:1). Seven altered pathways were selected and assessed based on pathway impact: GPL metabolism (essential for cell structure and signaling processes), linoleic and alpha-linolenic acid metabolism (involving important fatty acids), glycosylphosphatidylinositol-anchor biosynthesis (crucial for anchoring proteic molecules to cellular membranes), glycerolipid metabolism, PI signaling system (key signaling pathway) and last but not least arachidonic acid metabolism (involved in cellular growth but also inflammatory processes). The significant and consistent changes in these pathways and particular lipid molecules identified in the plasma of TC patients compared with healthy controls strongly suggest their value as serum markers for PTC diagnosis [12].

A research team led by Lu published a multi-omics investigation [18] on FA metabolism in differentiated thyroid cancer, combining metabolomic, lipidomic, and proteomic approaches on tumor and para-tumor tissue samples. The untargeted lipidomic analysis identified a significantly different lipid metabolome between the PTC group and the para-tumor group: while diacylglycerol levels were decreased, the levels of PL (such as PC, PI, phosphatidylglycerol, and cephalin) and SL (SM and CER) were notably increased in PTC, up to two or three times compared to the para-tumor group. This suggests that thyroid cancer is mainly influenced by lipid metabolism. Moreover, the multi-omics analysis provided evidence that all three stages of FA metabolism (hydrolysis, transportation, oxidation) are significantly enhanced in PTC tissue compared to para-tumor tissue. This was demonstrated by increased expression of key enzymes associated with these stages. Lipoprotein lipase (LPL), an important enzyme that is responsible for the hydrolysis of triglycerides into fatty acids, presented elevated expression at the level of tumor tissue. In addition to this, fatty acid transport protein 2 (FATP2), which plays an essential role in the uptake and transportation of FA into cells, was similarly upregulated. Carnitine palmitoyl transferase 1A (CPT1A), an enzyme involved in mitochondrial FA beta-oxidation, levels were increased as well. Essentially, the study established a direct correlation between increased expression of the three enzymes and tumor aggressiveness: elevated levels of LPL, FATP2, and CPT1A were associated with lymph node metastasis and more advanced tumor stages. In addition to this, elevated levels of FATP2 and CPT1A were independently linked to a dire prognosis, implying their role as prognostic markers of tumor progression [18]. These results actually demonstrate that thyroid cancer cells heavily rely on increased FA metabolism in order to satisfy their energetic and biosynthetic needs for proliferation and metastasis.

Ishikawa et al. published an IMS study on seven PTC cases, comparing cancerous tissue with healthy thyroid tissue. The main goal was to investigate the distribution and differences between the two types of tissue PL profiles. The IMS analysis was able to provide detailed spatial information regarding lipid distribution; several PL species were significantly higher within cancerous tissue compared to normal para-tumor tissue. The identification of these particular molecules was confirmed through tandem MS, and the regions of interest (ROI) were defined based on the corresponding hematoxylin and eosin-stained tissue sections. The results of the histological examination confirmed that cancerous areas showed classic features of PTC, including columnar epithelium displayed in papillary projections, respectively, a high cytoplasmic ratio and nuclear grooves and pseudo-inclusions. By contrast, normal thyroid tissue presented the characteristic follicles. The identified molecules were: PC (16:0/18:1), PC (16:0/18:2), and SM (d18:0/16:1). Elevated levels of these particular lipid species suggest an alteration of both cellular membrane composition and signaling pathways in neoplastic cells. IMS is a powerful diagnostic tool, with the correlation between the increase of specific PL in cancerous regions and thyroid cancer behavior suggesting their potential role as novel biomarkers for PTC diagnosis and prognosis [9].

In another IMS study, Wojakowska et al. analyzed tissue samples from three TC patients and compared cancerous to non-cancerous thyroid tissue lipid profiles. After the ROI were defined by a pathologist for each tissue specimen, the samples were submitted for analysis, and then the ROI pairs were compared for each patient, and between the three patients as well. The average number of molecules differentiating paired tumor and para-tumor tissue was 229, while the average number of differentiating components among cancerous regions was 232; for the non-cancerous ROIs, the average number of discriminating molecules was 281. These findings suggest a higher similarity between PTC tissue originating from different patients, compared to non-cancerous tissue from different individuals. Moreover, when they analyzed the overlap of PTC-upregulated and -downregulated molecules of all three patients, 23 commonly downregulated and 33 commonly upregulated components were identified. Among the upregulated lipid species, eight had statistically significant elevated levels in PTC tissue: PC (32:0), PC (32:1), PC (34:1), PC (36:3), PA (36:2), PA (36:2), SM (34:1) and SM (36:1); these could be used in clinical practice to discriminate between malignant and benign thyroid pathology and/or healthy patients [15].

Lee et al. conducted a comprehensive study using nanoflow ultrahigh-performance liquid chromatography-electrospray ionization-tandem mass spectrometry (nUHPLC-MS/MS), investigating the lipid profiles of patient plasma samples for five different types of cancer, and compared them with healthy controls. Among others (liver, lung, colorectal, and gastric cancers), this study group included 10 patients with TC. The results showed that numerous lipids were significantly and simultaneously modified in at least two types of neoplasm, but few species were unique for a particular type. However, TC presented a distinct "fingerprint" with the following characteristics: a high-abundance PE species (38:4, 38:3, and 40:6) was significantly decreased, while lysoPI 18:0 and 18:1 levels were notably higher. If the other four types of cancer included in the study had high-abundance lysoPE and PE plasmalogen species commonly decreased, by contrast, TC had particular species, such as lysoPE 18:1, 18:2, and PE 16:1p/22:6, increased. SM d18:1/22:0 was also decreased by approximately two-fold in the serum of PTC patients. The conclusion of this research emphasizes the need for further studies, with a larger number of patients/samples, in order to establish and validate cancer-specific lipid biomarkers [6] (Table 1).

**Table 1:** Reviewed publications and main findings IMS: Imaging mass spectrometry; UPLC-Q-TOF-MS: ultra-performance liquid chromatography quadrupole time-of-flight mass spectrometry; UHPLC-QTOF-ESI-MS: ultra-high-performance liquid chromatography quadrupole time-of-flight electrospray ionization mass spectrometry; HPLC-MS: high-performance liquid chromatography mass spectrometry; UPLC-MS: ultra-performance liquid chromatography mass spectrometry; nUHPLC-MS/MS: nanoflow ultrahigh performance liquid chromatography-tandem mass spectrometry; PC: phosphatidylcholine; SM: sphingomyelin; PTC: papillary thyroid carcinoma; PL: phospholipid; GPL: glycerophospholipid; SL: sphingolipid; C: control; PGA: prostaglandin A; PGE: prostaglandin E; B: benign; FA: fatty acid; LPL: lipoprotein lipase; FATP2: fatty acid transport protein 2; PG: prostaglandin; CPT1A: carnitine palmitoyl transferase 1A; PA: phosphatidic acid; PE: phosphatidyletanolamine; lysoPI: lysophosphadidylinositol; lysoPE: lysophosphatidyletanolamine

Authors	Title	Year	Design	Population	Sample type	Investigation method	Findings
Ishikawa et al. [9]	Increased expression of phosphatidylcholine (16:0/18:1) and (16:0/18:2) in thyroid papillary cancer	2012	Case series	7 patients	Tissue	IMS	PC 16:0/18:1 and 16:0/18:2, and SM d18:0/16:1 are significantly increased in PTC compared to control group.
Wojakowska et al. [15]	Discrimination of papillary thyroid cancer from non-cancerous thyroid tissue based on lipid profiling by mass spectrometry imaging	2018	Case series	3 patients	Tissue	IMS	8 PL species have significantly higher levels in PTC and can be used to discriminate malignant from benign or healthy tissue.
Jiang et al. [12]	Plasma lipidomics profiling reveals biomarkers for papillary thyroid cancer diagnosis	2021	Case control	80 patients	Plasma	UPLC-Q-TOF-MS	13 GPL and SL species showed significant differences between PTC and C group.
Berinde et al. [17]	Metabolic profiles and blood biomarkers to discriminate between benign thyroid nodules and papillary carcinoma, based on UHPLC-QTOF-ESI+-MS analysis	2024	Case control	136 patients	Serum	UHPLC-QTOF-ESI-MS	Oleic acid derivatives, dihydrocortisol and PGA and PGE discriminate well between PTC and B.
Berinde et al. [16]	In search of relevant urinary biomarkers for thyroid papillary carcinoma and benign thyroid nodule differentiation, targeting metabolic profiles and pathways via UHPLC-QTOF-ESI+-MS analysis	2024	Case control	80	Urine	UHPLC-QTOF-ESI-MS	Lipidomic window proved most relevant for finding PTC biomarkers; revealed alterations in free FA metabolism steroid hormones, PL and PG.
Lu et al. [18]	Multi-omics analysis of fatty acid metabolism in thyroid carcinoma	2021	Case control	48 patients	Tissue	HPLC-MS	FA metabolism is significantly enhanced in PTC; elevated expression of LPL, FATP2 and CPT1A was linked to tumor aggressiveness.
Wang et al. [14]	Combined metabolomic and lipidomic analysis uncovers metabolic profile and biomarkers for papillary thyroid carcinoma	2023	Case control	194	Plasma	UPLC-MS	Lipid metabolism was increased. PA, PE and SM relative concentrations were increased in PTC. FA oxidation was downregulated.
Lee et al. [6]	Plasma lipid profile comparison of five different cancers by nanoflow ultrahigh performance liquid chromatography-tandem mass spectrometry	2019	Case control	104	Plasma	nUHPLC-MS/MS	Increased levels in lysoPI 18:0 and 18:1, and lysoPE 18:1 and 18:2 proved to be specific for PTC.

Discussion

This systematic review aims to emphasize the growing body of evidence proving the importance of lipid metabolism in thyroid cancer. Several studies have revealed consistent alterations in various lipid classes across different types of samples (serum, plasma, urine, or tissue) [6,9,12,14-18]. These changes play an essential part in the complex metabolic reprogramming responsible for the development and growth of TC.

Lipidomics has been used in cancer-related research, which has confirmed the nature and the specifics of lipid changes within tumor microenvironment [19]. FA metabolism in PTC was also investigated by a research team led by Liu who observed that pyruvate carboxylase, an essential enzyme involved in the tricarboxylic acid cycle, was overexpressed in TC, consequently increasing FA synthesis within tumoral tissues. Increased expression was correlated with more aggressive features of PTC such as lymph node metastasis and tumor invasion. This emphasizes the role of impaired FA metabolism in tumor behavior, suggesting the therapeutic potential that targeting this metabolic pathway may have [20].

There is evidence suggesting that while PL and SL levels are elevated in PTC, the relative concentration of FA and acylcarnitine is, by contrast, decreased, indicating a downregulated FA oxidation and increased lipid metabolism [14]. FA oxidation is a major energy source in the mitochondria, where long-chain FA are converted to acyl coenzyme A and then to acylcarnitine by CPT1A. Studies showed that FA oxidation acts differently in different types of tumors: it is upregulated in Kirsten rat sarcoma viral oncogene mutant lung, liver, and breast cancer [21]; by contrast, FA oxidation is downregulated in lymphoma and excess FA oxidation-derived adenosine triphosphate (ATP) seems to inhibit leukemia cell survival. Furthermore, FA oxidation activation enhances the aggressive behavior of ovarian cancer, increasing its metastatic potential [22]. As a well-differentiated tumor with favorable mid- and long-term prognosis, PTC may show different metabolic characteristics and biological behavior than other types of cancer, both differentiated and undifferentiated.

Tumor development and progression involve complex interactions and multiple processes, including cell proliferation, apoptosis, angiogenesis, and metastasis, as well as particular conditions within tumor microenvironment such as hypoxia, increased tolerance to reactive oxygen species and impaired immunity; all of these profoundly influence the primary metabolic pathways [23-25]. As far as lipid metabolism is concerned, tumor metabolites are defined by increased levels of lipid molecules, which are crucial for the construction of cell membranes. PL are major components of cell membranes that ensure the plasticity and fluidity of cell, and in this light, any alterations in membrane PL may critically influence tumor phenotypes [26,27]. PE, for instance, is essential for maintaining cell membrane fluidity and studies [6,12] have revealed that several species (PE 36:1, PE 36:3, PE 38:6, PE 18:0/20:4, PE-Nme 18:1/18:1) were upregulated in PTC patients compared to controls, while others, by contrast, were downregulated in cancer patients. PE has been shown to be closely linked to calcium transport regulation, which is highly important considering that in TC cells, calcium transport remodeling facilitates cell proliferation and invasion [28,29].

Previous studies have demonstrated that modified PC distribution may alter the cellular lipid membrane microenvironment, consequently leading to impaired membrane function. This probably accounts for the increased PC levels in rapidly growing cancer cells [27,30]. Atypical PC distribution has been observed not only in TC patients, but in various types of neoplasms, including breast, lung, colorectal, gastric, and oral tumors [30-32]. Several lipid molecules including PC (16:0/18:1), PC (16:0/18:2 ), PC (32:0), PC (32:1), PC (34:1) PC (36:3), SM (d18:0/16:1), SM (34:1), and SM (36:1), have shown significantly increased levels in PTC tissue compared to normal tissue; this indicates these lipid classes are directly linked to TC pathogenesis and they may serve as reliable biomarkers for differentiating between PTC patients and healthy individuals [9,15,33]. At a cellular level, PC is mediated by phospholipase A2 (PLA2), an enzyme responsible for hydrolyzing GPL to FA and lysoPC. The enzyme is notably more active in TC tissue compared to normal tissue. Hence, PC and its metabolites play an essential part in tumor growth and survival [34].

There is no doubt that the existing studies have certain limitations, such as small study groups, heterogeneity in lipidomic platforms, lack of standardized protocols, the underrepresentation of follicular and undifferentiated tumors, the absence of studies concomitantly analyzing different types of samples originating from the same patient (blood, urine, tissue), and also the risk of publication bias. However, as Farrokhi et al. emphasized, research using "omics" approaches could contribute to the discovery, in the near future, of new biomarkers for thyroid carcinoma and improve diagnosis, prognosis, and help develop more effective therapies for thyroid cancer and allow for more personalized treatment strategies [13].

## Conclusions

There is no doubt that lipidomics is a burgeoning field that holds tremendous potential in terms of diagnostic and therapeutic options for cancer patients. The reviewed studies consistently revealed significant changes in the metabolic and lipidomic profiles associated with differentiated thyroid cancer, using various samples (urine, plasma, serum, and tissue). A recurring aspect among all research is the profound dysregulation of lipid metabolism, encompassing various lipid classes (PL, SL, PG, etc.) and signaling pathways (linoleic and α-linolenic acid, steroid hormones, arachidonic acid, etc.) within cancer cells. Elevated levels of different PL and SL species, along with decreased acylcarnitines and FA levels, suggest there is an increased metabolism and altered FA synthesis in PTC patients. Additionally, the upregulation of LPL, FATP2, and CPT1A, which are essential enzymes involved in FA metabolism, is directly correlated with tumor aggressiveness and lymph node metastases, making these viable biomarkers for PTC prognosis.

The findings from already published lipidomics research have provided important insights into the role of lipids in thyroid tumorigenesis and revealed promising novel biomarkers. Even so, further research, especially large-scale prospective multicenter studies, using standardized methods for sample collection, data normalization, and shared databases, is needed to validate these results and put them to use in everyday practice. Hence, there is a need for developing standardized protocols for lipidomic analysis to improve data reproducibility and, of course, comparability across studies. Future research directions in this field should include the integration of lipidomics with other omics data (such as proteomics and genomics) to provide an even more comprehensive understanding of the mechanisms involved in thyroid neoplasm physiopathology. Moreover, we should focus on investigating the role of lipidomics in TC stem cells, as well as the tumor microenvironment. Finally, one of the most important directions is, undoubtedly, the exploration of the therapeutic potential of lipid metabolism.
